# Statistical controversies in clinical research: long-term follow-up of clinical trials in cancer

**DOI:** 10.1093/annonc/mdv392

**Published:** 2015-10-03

**Authors:** J. Cuzick

**Affiliations:** Centre for Cancer Prevention, Wolfson Institute of Preventive Medicine, Queen Mary University of London, London, UK

**Keywords:** clinical trials, follow-up

## Abstract

Long-term follow-up is an important unmet need for the full analysis of new treatments for cancer. Earlier detection of cancer and more effective treatment have led to many more patients surviving for more than 5 and even 10 years, so that evaluating late recurrences and side-effects is an increasingly important issue. This is particularly relevant for oestrogen receptor-positive breast cancer, where the existence of late recurrences is well documented. However, survival for other cancers, notably prostate, colorectal and cervix cancer, has dramatically increased in recent years due to screening and better treatment of early lesions. Trials of preventive therapies have an even greater need for long follow-up. Here, we review these issues and suggest ways in which provision for long-term follow-up can be improved.

## Abstract (precis)

Individuals participating in a clinical trial have a right for their experiences to be fully analysed. Cancer is generally being diagnosed earlier and treatments are more effective, so long-term outcomes are increasingly important. Provision for long-term follow up needs to be made at the outset for trials. Patient advocate groups have an important role to play in facilitation of this activity.

## introduction

**Figure 1.**
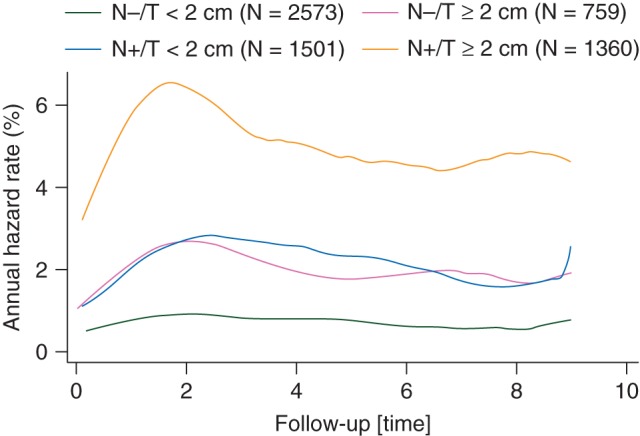
Annual hazard rates for distant recurrence in the ATAC trial according to nodal status (positive/negative) and tumour size (<2 versus ≥2 cm) in the first 10 years of follow-up.

The prognosis of many cancers has dramatically improved over the past few decades, so that a much larger proportion of patients are experiencing an extended post-treatment period without recurrent disease. This is particularly true for breast cancer, where most of my experience lies. For example, In the UK, there were 49 946 new cases of breast cancer in 2011 and 16 067 deaths from it in 2012, indicating that just over two thirds of women diagnosed with breast cancer will live long enough to die of something else. One consequence of this is that disease-free survival and overall survival are no longer as useful as they once were for assessing treatment effectiveness. For these end points, the contribution from disease-related events is very much diluted by deaths from causes unrelated to disease progression or treatment side-effects. This is discussed more fully in ref. [1]. Another consequence of generally better prognosis is that control of late recurrence is an increasingly important goal of treatment. A peak in recurrence rates around year 2 for oestrogen receptor-positive breast cancer had been seen in older studies, but this is very much diminished in more recent studies, where now the recurrence rate is roughly constant for at least 10 years (Figure 1). Evidence from earlier studies indicates that this roughly constant rate of recurrence continues for at least another 10 years after this [2, 3].

## late recurrence in breast cancer

This is generally good prognosis but a continuing rate of late recurrence in breast cancer has led to an interest in extending adjuvant hormonal treatment from 5 to 10 years. A real but modest reduction in recurrence rates seen in the ATLAS and ATTOM trials for 10 years of tamoxifen compared with 5 years of treatment [4, 5], but this was not apparent until after 10 years of follow-up. This was due to the ‘carryover’ effect of 5 years of treatment leading to a reduction in recurrence in years 5–10 associated with the first 5 years of treatment so that a minimum of 15 years of follow-up was needed to see this important effect. Mortality differences will take even longer to establish, and 20 years of follow-up will be necessary to obtain a clearer picture of the impact of extended tamoxifen treatment on the death rate from breast cancer.

More recently, a number of trials have compared an aromatase inhibitor to tamoxifen in a range of situations [6]. Long-term follow-up of these trials is essential to get a clear picture about the overall benefits of aromatase inhibitors versus tamoxifen. In the ATAC trial of 5 years of treatment with either tamoxifen or anastrozole, a full comparison of the benefits and risks is not yet available as only 10-year follow-up results have been reported [7]. At that time, the recurrence curves were still separating, and the ratio of recurrence and distant recurrence rates between treatment arms at year 10 was similar to earlier times with about a 15% reduction for anastrozole (Figure 1) indicating that the total benefits and total number needed to treat with anastrozole versus tamoxifen to prevent one recurrence may very well be substantially underestimated at this point. However, the impact on breast cancer mortality was not significant at this stage, although the effect on distant recurrence was established, indicating the need for further follow-up to see if this will be achieved. In 2009, we undertook a post 10-year long-term follow-up of the ATAC trial under the name of Long-term Anastrozole versus Tamoxifen Treatment Effects (LATTE). To avoid much of the unnecessary reporting requirements needed for a CTIMP trial, given that patients were no longer receiving active treatment, we took the approach of formally closing the ATAC trial as a clinical trial and reopening it as an epidemiologic long-term follow-up study. This has many advantages in terms of streamlining reporting requirements in patients who have been off treatment for at least 5 years. Our initial goal is to report 15-year outcome and an adequate follow-up interval is now available to do this. We have focused on the two monotherapy arms, where there is the greatest clinical interest (tamoxifen alone versus anastrozole alone for 5 years). Follow-up in the UK, where 36% of the eligible patients were recruited has been satisfactory with recurrence and mortality data obtained for more than 75% of 1513 eligible patients (i.e. alive after 10 years of follow-up). High participation has also occurred in Australia and New Zealand. However, ATAC was an international study with recruitment from 381 centres in 21 countries. The trial remains largely blinded, so that additional follow-up will be very informative and unbiased. In Europe, follow-up has been mixed with slow, but reasonable results in the Netherlands, France, Italy and Sweden, but much poorer in some countries—notably Spain in which the Regulatory Authorities have insisted that this must be conducted as a CTIMP trial with all the attendant requirements and paperwork. Progress has also been very poor in the United States, where 22% of the patients eligible for post 10-year follow-up were entered. This is despite regular contact and the support of the American Cancer Society. To date, we have only received follow-up information for 5% of the 1025 eligible women. The reasons for this are many—including requests for large financial payments, although compensation which more than covered the time to look up follow-up records was offered, unreasonable demands from ethics committees for a new ethics approval with which included large payment requests and further consent, incomplete long-term follow-up records at the clinics for women in this trial due partly to discharging women after 10 years of follow-up.

## continued long-term treatment

Trials of the addition of aromatase inhibitors after 5 years of tamoxifen have also shown important recurrence reductions [8], but again the full benefit may not as yet have been observed and crossover of the control arm to receive an AI makes this difficult to evaluate [9]. I also think it is premature to use these trials to make conclusions about the most appropriate duration of adjuvant treatment with an aromatase inhibitor, as duration of AI treatment *ab initio* is clearly different from switching to an AI after several years of tamoxifen treatment. This distinction is clearly made in the ASCO guidelines [10] and by others [11], but many clinicians claim that use of AIs for more than 5 years is appropriate in some patients.

Long-term follow-up is also essential to document side-effects and harms related to treatment. An important example is the increase in myocardial infarction and cardiac death after radiotherapy from breast cancer, which only emerged after 10 years of follow-up and was the major reason why the early radiotherapy trials did not show an impact on overall survival [12–14]. Discovery of these late toxicities has led to a major change in radiotherapy planning and delivery, and the prospect from reduced cardiac toxicity and improved overall mortality using this modality is now high [15].

Another important example is the use of tamoxifen where the continued occurrence of endometrial cancer after treatment completion is well documented in the adjuvant treatment setting [16, 17], but seems to be less clear in the prevention setting [18], possibly because of the younger age and greater number of premenopausal women recruited into these studies.

## preventive therapy

The importance of long-term follow-up is even more central to the full evaluation of preventive agents in breast cancer, where there has been the most activity. Many of the trials have been stopped and unblinded at an early stage when a reduction in breast cancer incidence was observed, but follow-up was not sufficiently mature to make a full assessment of benefits and risks. This has had a detrimental impact on the implementation of preventive therapies such as tamoxifen and raloxifene, despite the fact that they have been licensed for this use in the United States by the FDA and in the UK by NICE (National Institute for Health and Care Excellence). The feasibility and implementation of continued follow-up is well illustrated by the IBIS-I tamoxifen prevention trial. This trial began in 1992 and first results were published in 2003 [19], showing a clear benefit on breast cancer incidence. All women in this trial were informed of this result. Most women agreed to remain blinded to their individual treatment and to continue follow-up. As a result, we were able to obtain reliable information and report the results after a median of 8 years [20] in 2007 when all treatment was completed, and again after 16 years median follow-up [18], when truly long-term effects began to be apparent. At that time, more than 75% of women who have not developed cancer still remained blinded. This last report showed an unexpected continued benefit of tamoxifen on recurrence which was unabated after 20 years of follow-up at around a 30% reduction in new breast cancers per year, so that 99 cancers had been prevented in this study by this time, and the number needed to treat with tamoxifen for 5 years to prevent one breast cancer fell from 55 at 10 years to 22 after 20 years of follow-up. However, mortality data are still immature as only 5% of the women in this trial have died (median age now is 66 years) and only 10% of women who developed breast cancer have died from it. Thus, another 10 years of follow-up will be needed to reliably assess impact of prophylactic tamoxifen or death from breast cancer. Fortunately, in the UK, this information is available from passive follow-up using national registries, but this remains a major challenge for Australia where 37% of the patients were recruited, but no national death index exists.

It is unfortunate that the similar NSABP–P1 trial unblinded their patients at a median follow-up time of 48 months [21] so that an unconfounded comparison after that period was not possible, although the 2005 report [22] did show the continuing low rate of breast cancer in the tamoxifen arm up to 7 years. Even more unfortunate is the unblinding of the only prevention trial using the aromatase inhibitor exemestane after only 35 months median follow-up when a large 65% reduction in invasive breast cancer was seen, based on 11 versus 32 events [23]. All women were informed of which arm to which they were randomized, and no subsequent follow-up has been reported. Indeed, this may very well not be done, due to the lack of funding from the pharmaceutical company who supported this trial. As a result, this has limited the interest of using this attractive drug for prevention, although the lack of company support to licence it for this indication has also been an issue.

Similar issues also arise for prostate cancer prevention, where two large studies of 5α-reductase inhibitors have been conducted [24, 25] and long-term follow-up is essential to interpret the early increase of high-grade cancers with these drugs.

## other cancers (non-breast)

While late recurrence is a particularly important aspect of oestrogen receptor-positive breast cancer, it is also important for other cancers with a relatively good prognosis—notably screen-detected prostate and colorectal cancers. A good example of the need for continued long-term follow-up is clinically localized prostate cancer where the death rate is virtually constant for 20 years and we have found that a cell-cycle progression score can usefully predict the risk of prostate cancer death in both the 0–5 and 5–10 years follow-up period [26], and potentially longer, although the data do not yet exist for this. Studies of preventive therapy for prostate cancer and aspirin prophylaxis of colorectal, stomach, oesophageal, breast, prostate and lung cancer will also require very long follow-up [24, 27, 28].

## potential solutions

A range of measures to address these problems exist, and their feasibility will depend on the medical infrastructure where the trial is being conducted. Some options are outlined in Table 1. The potentially easiest, and most important, is to establish links with national death indices so that at least cause and date of death can be obtained. Such registries exist in most countries, but a major obstacle has been the difficulties in accessing this information arising from data protection and other administrative requirements. There is no need for these obstacles to exist, provided consent for long-term follow-up is given by the patient at the onset of the trial.

**Table 1.** Potential methods for improving long-term follow-up in clinical trials

Action	Utility	Issues
Greater use of national death registries	Mortality most important longer term event for many trials	No information on recurrence/progression or side-effects
Centralized hospital record systems	Ideal for most purposes	Not widely available. Data access issues difficult when they are
Better links with family doctors	Only medical contact after discharge from specialist	Can be difficult to engage. Not helpful if patient changes doctor.
Use of social media	Maintains direct contact with the patient	Events often require validation by doctor/nurse
Identifying close friend/relative in case contact in lost with patient	Fall back if patient contact lost	May also not be contactable

A better, but less often available approach is to link up with hospital records via regional/national hospital registry databases. In some countries, notably in Scandinavia and the UK, this can be now achieved in a relatively straightforward way, using passive follow-up through national registries and record systems. However, in the UK, a remaining main obstacle has been the excessive requirements and attendant delays involved in obtaining these data. In most other countries, such systems do not exist, but the increasing use of computerized record systems suggests that this might be available more in the future. A currently more viable approach in some areas is to establish closer links with the patient's general practitioner/family doctor or group health practitioner. These links with clinical trials are often weak or non-existent, but can potentially provide useful information once the patient has been discharged from specialist care. Maintaining this contact can be a challenge and GPs generally do not feel an obligation to support clinical trials, but this can be an important source of information, and keeping GPs more informed about clinical trials is important for this and many other reasons. Of course, patients will move or change doctors and involving the new GP is a greater challenge.

Another approach is to involve the patient directly in the long-term follow-up process. This has proven to be very important in our breast cancer prevention trial, where we sent participants a (roughly) annual report on the progress and at the same time asked them about recurrence and any late side-effects. This was done by post, but with the advent of widespread email/mobile phone use, much of this can now be done more efficiently using social media and relying on the post only in special occasions. We have found that collecting contact details on a close relative or friend can also be useful when direct contact has been lost, e.g. if the patient has died.

## conclusions

Individuals participating in a clinical trial have a right for their experiences to be fully analysed, and the ethical justification for impediments to long-term follow-up urgently needs to be addressed by the medical community. Provision for some sort of long-term follow-up needs to be made at the outset for trials, and this should be a requirement for their approval. Ideally, all trial patients would be followed until death. However, the need for long-term follow-up will depend to some extent on the type of trial and the environment in which it is being conducted. For trials of metastatic disease or other high mortality situations, most of the potential information will have accrued after 5 years or so of follow-up. However, cancer is generally being diagnosed earlier and treatments are more effective so long-term outcomes are increasingly important. In some countries, this can be achieved using passive follow-up through national registries and record systems. Elsewhere, we need to be more creative and learn how to exploit social media to keep in contact with trial patients directly. Patient advocate groups have an important role to play in facilitating this activity. We also need to be more cautious about universal unblinding of trials when the initial end points are met. In our experience, by treating patients as partners in our clinical studies, keeping them informed about trial results via an (annual) newsletter and explaining the need to continue to keep trial follow-up active long after treatment is completed to obtain definitive results, we have found few patients request to be unblinded at an early stage. Where possible this has enabled us to provide a more complete picture about the full long-term impact of new treatments.

## disclosure

The author has declared no conflicts of interest.
